# Enhancement of the serum chloride concentration by administration of sodium–glucose cotransporter-2 inhibitor and its mechanisms and clinical significance in type 2 diabetic patients: a pilot study

**DOI:** 10.1186/s13098-020-0515-x

**Published:** 2020-01-13

**Authors:** Hajime Kataoka, Yuichi Yoshida

**Affiliations:** 1 Internal Medicine, Nishida Hospital, 2-266 Tsuruoka-Nishi-machi, Saiki, Oita 876-0047 Japan; 2 Department of Endocrinology, Metabolism, Rheumatology and Nephrology, Faculty of Medicine, Oita University, 1-1 Idai-ga-oka, Hasama-machi, Yufu, Oita 879-5593 Japan

**Keywords:** Sodium–glucose cotransporter-2 inhibitor, Chloride, RAAS, Ketone

## Abstract

**Background:**

Chloride is a key electrolyte that regulates the body fluid distribution. Accordingly, manipulating chloride kinetics by selecting a suitable diuretic could be an attractive strategy for correcting body fluid dysregulation. Therefore, this study examined the effects and contributing factors of a sodium–glucose cotransporter-2 inhibitor (SGLT2i) on the serum chloride concentration in type 2 diabetic (T2DM) patients without heart failure (HF).

**Methods:**

This study was a retrospective single-center observational study that enrolled 10 T2DM/non-HF outpatients for whom the SGLT2i empagliflozin (daily oral dose of 10 mg) was prescribed. Among these 10 patients, 6 underwent detailed clinical testing that included hormonal and metabolic blood tests.

**Results:**

Empagliflozin treatment for 1–2 months decreased body weight (− 2.69 ± 1.9 kg; p = 0.002) and HbA1c (− 0.88 ± 0.55%; p = 0.0007). The hemoglobin (+ 0.27 ± 0.36 g/dL; p = 0.04) and hematocrit (+ 1.34 ± 1.38%; p = 0.014) values increased, but the serum creatinine concentration remained unchanged. The serum chloride concentration increased from 104 ± 3.23 to 106 ± 2.80 mEq/L (p = 0.004), but the sodium and potassium concentrations did not change. The spot urinary sodium concentration decreased from 159 ± 43 to 98 ± 35 mEq/L (p < 0.02) and the spot urinary chloride tended to decrease (from 162 ± 59 to 104 ± 36 mEq/L, p < 0.08). Both renin and aldosterone tended to be activated (5/6, 83%). The strong organic acid metabolite concentrations of serum acetoacetate (from 42 ± 25 to 100 ± 45 μmol/L, p < 0.02) and total ketone bodies (from 112 ± 64 to 300 ± 177 μmol/L, p < 0.04) increased, but the actual HCO_3_^−^ concentration decreased (from 27 ± 2.5 to 24 ± 1.6 mEq/L, p < 0.008).

**Conclusions:**

The present study demonstrated that SGLT2i enhances the serum chloride concentration in T2DM patients and suggests that the effect is mediated by the possible following mechanisms: (1) enhanced reabsorption of urinary chloride by aldosterone activation due to blood pressure lowering and blood vessel contraction effects, (2) reciprocal increase in the serum chloride concentration by reducing the serum HCO_3_^−^ concentration via a buffering effect of strong organic acid metabolites, and (3) reduced NaHCO_3_ reabsorption and concurrently enhanced chloride reabsorption in the urinary tubules by inhibiting Na^+^–H^+^ exchanger 3 in the renal proximal tubules. Thus, the diuretic SGLT2i induces excessive extravascular fluid to drain into the vascular space by the enhanced vascular “tonicity” caused by the elevated serum chloride concentration.

## Background

Diabetes mellitus is a worldwide public health and economic problem. Glucose-regulating drug of sodium–glucose cotransporter 2 inhibitor (SGLT2i) may offer unique benefits as glucose-lowering agent [1–3]. Importantly, SGLT2i appear to have pleotropic effects [1–3] that are also cardioprotective and renal protective in patients with type 2 diabetes mellitus (T2DM) [4–6], and are now widely approved as antihyperglycemic agents.

Recent studies demonstrated that chloride is a key electrolyte for regulating plasma volume during worsening heart failure (HF) [7] and its recovery [8], leading to the development of the “chloride theory” for HF pathophysiology [9, 10] and a diuretic treatment strategy by modulating the serum chloride concentration [10, 11]. According to the “chloride theory” [10], dysregulated body fluid distribution can be adjusted by manipulating the serum chloride concentration using a suitable diuretic. SGLT2i is reported to exert diuresis through osmotic and natriuretic effects that contribute to plasma contraction and decrease blood pressure [1–3]. There is a paucity of data, however, regarding the effects of this agent on the serum chloride concentration, and even less is known about its precise diuretic mechanisms. Therefore, this study investigated the effects and possible underlying mechanisms of diuretic of an SGLT2i on the serum chloride concentration and its clinical significance according to the “chloride theory” [10] in T2DM/non-HF patients.

## Methods

### Study design

This study was a retrospective single-center observational study that enrolled outpatients with T2DM/non-HF at Nishida Hospital (Saiki-city, Oita, Japan) prescribed empagliflozin (daily oral dose of 10 mg) between March 2017 and April 2018. The effects of empagliflozin on the serum chloride concentration were evaluated 1 to 2 months after administration was initiated. During the study period, empagliflozin was prescribed to 20 T2DM/non-HF patients, but 10 patients were excluded from the present study due to the lack of required clinical data (n = 6), complications with a serious co-morbidity requiring hospitalization (n = 2), or use of a diuretic (n = 2). The 10 remaining clinically stable T2DM/non-HF patients were the subjects analyzed in the present study. Among these 10 patients, 6 underwent detailed clinical examinations, including hormonal and metabolic blood tests, to estimate the factors contributing to the SGLT2i-mediated modulation of the serum chloride concentration. None of the patients enrolled in the study had used diuretics other than SGLT2i either before the initiation of SGLT2i administration or throughout the study period of SGLT2i treatment evaluation.

The ethics committee at Nishida Hospital approved the study protocol. Written informed consent was obtained from all patients before study enrollment.

### Data collection and analytic methods

Physical examination, blood tests of peripheral venous blood (hematologic, diabetic/metabolic, b-type natriuretic peptide, venous blood gas, and neurohormonal tests), and a spot urine test for electrolytes were performed at baseline and 1 to 2 months after beginning SGLT2i administration. The blood samples were obtained after patients rested in a supine position for 20-min. Peripheral blood tests, analyzed by standard techniques, included a red blood cell count, hemoglobin, hematocrit, mean red blood cell volume, albumin, serum and urinary electrolytes (sodium, potassium, and chloride), blood urea nitrogen, and creatinine. The spot urine test included measurement of electrolyte concentrations. Hemoglobin A1_c_ (%) was determined by high performance liquid chromatography. Plasma b-type natriuretic peptide was measured by chemiluminescent immunoassay. Plasma renin activity was measured by enzyme immunoassay. Aldosterone and antidiuretic hormone levels were measured by radioimmunoassay. Plasma acetoacetate, 3-hydroxybutyrate, and total ketone body concentrations were measured by the enzyme cycling method. Total CO_2_, actual HCO_3_^−^, and pO_2_ were analyzed using an ABL80 FLEX autoanalyzer (Radiometer Medical ApS, Brφnshφj, Denmark).

### Statistical analysis

All statistical analyses were performed using GraphPad Prism 4 (San Diego, CA). All data are expressed as the mean ± SD for continuous data and percentage for categorical data. Paired *t* tests for continuous data were used for two-group comparisons. All tests were 2-tailed, and a p value < 0.05 was considered statistically significant.

## Results

The clinical characteristics of the 10 T2DM/non-HF patients at study entry are shown in Table 1. Mean patient age was 61.7 ± 14 years (range, 36–92 years), and 40% were men. Cardiac function and renal function were preserved in all study patients, determined mainly on the basis of the b-type natriuretic level and estimated glomerular filtration rate. One patient had a history of myocardial infarction. None of the patients were on diuretics. Therapy for diabetes included isolated or combined use of metformin, sulfonylureas, and/or dipeptidyl peptidase-4 inhibitors.

**Table 1:** Clinical characteristics of the study patients at baseline

Characteristics	n = 10
Age, years (mean ± SD)	61.7 ± 14 (36–92)
Male	4
Body mass index (kg/m^2^)	32.1 ± 3.7 (28.2–40)
eGFR (mL min^−1^·1.73 m^−2^)	76.3 ± 14.4 (51–90)
Cardiovascular disease	
Hypertension	7
Hyperlipidemia	6
Ischemic heart disease	1
Medication for cardiovascular disease	
ACE inhibitors/ARB	5
Beta-blockers	2
Calcium antagonists	5
Nitrate	1
Diuretics	0
Lipid-lowering drugs	7
Therapy for diabetes	
Metformin	5
Sulfonylureas	2
Dipetidyl peptidase-4 inhibitors	7
Thiazolidinediones	0
Glucagon-like peptide-1 antagonist	0
Insulin	2

Data presented as number (%) of patients otherwise specified. *eGFR* estimated glomerular filtration rate, *ACE* angiotensin-converting enzyme, *ARB* angiotensin II receptor blocker

SGLT2i treatment details for the six study patients are presented in Table 2. After 1 to 2 months (34.9 ± 7.4 [range 28–53] days) of empagliflozin administration, body weight (− 2.69 ± 1.9 [range, − 6.1 to − 0.2] kg; p = 0.002) and hemoglobin A1_c_ (− 0.88 ± 0.55 [range, − 1.8 to − 0.2]%; p = 0.0007) decreased. Serum b-type natriuretic peptide levels did not change. Hemoglobin (+0.27 ± 0.36 [range, − 0.5 to +0.8] g/dL; p = 0.04) and hematocrit (+ 1.34 ± 1.38 [range, − 0.7 to +4.1]%; p = 0.014) increased, but the serum creatinine concentration was unchanged. The serum chloride concentration increased from 104 ± 3.23 to 106 ± 2.80 mEq/L with a mean change of 2.1 ± 1.7 mEq/L (range, − 1 to + 5 mEq/L; p = 0.004), but the serum sodium and potassium concentrations did not change. The spot urine test revealed that the sodium concentration decreased (from 159 ± 43 to 98 ± 35 mEq/L, p < 0.02) and the chloride concentration tended to decrease (from 162 ± 59 to 104 ± 36 mEq/L, p < 0.08). Both renin and aldosterone tended to be activated (5/6 patients, 83%). Serum levels of strong organic acid metabolites of acetoacetate (+ 57.8 ± 38.5 μmol/L; range, + 7 to + 107 μmol/L; p < 0.02) and total ketone bodies (+ 188 ± 167 μmol/L; range, − 17 to + 470 μmol/L; p < 0.04) increased, but the serum levels of actual HCO_3_^−^ decreased (− 2.5 ± 1.4 mEq/L; range, − 4.2 to − 0.8 mEq/L; p < 0.008).

**Table 2:** Changes in physical and blood test after sodium-glucose transporter-2 inhibitor treatment in type 2 diabetic patients without heart failure

	Normal range	Before	After	After vs. before (increase/unchanged/decrease)	p value
Physical examination (n = 10)					
Body weight (kg)		80.4 ± 17.5	77.8 ± 17.3	0/0/10	0.002*
Blood pressure (mmHg)					
Systolic pressure		128 ± 9.83	121 ± 13.5	2/0/8	0.11
Diastolic pressure		75.8 ± 9.0	75.6 ± 9.4	4/0/6	0.96
Heart rate (bpm)		68.8 ± 11.4	70.3 ± 13.5	6/0/4	0.59
Diabetes-related blood test (n = 10)					
HbA1_C_ (%)	4.6–6.2	8.55 ± 0.94	7.67 ± 0.86	0/0/10	0.0007*
Fasting blood glucose (mg/dL)	73–109	179 ± 40.5	138 ± 18.4	1/0/9	0.006*
LDL cholesterol (mg/dL)	63–163	106 ± 18.5	97.6 ± 22.4	3/0/7	0.07
HDL cholesterol mg/dL)	48–103	47.7 ± 8.7	47.3 ± 9.7	8/0/2	0.86
B-type natriuretic peptide (pg/mL) (n = 6)	< 18.4	8.8 ± 3.75	12.0 ± 9.35	2/2/2′	0.47
Peripheral blood test (n = 10)					
Hemoglobin (g/dL)	11.6–14.8	13.9 ± 1.29	14.1 ± 1.19	8/1/1′	0.04*
Hematocrit (%)	35.1–44.4	41.0 ± 3.51	42.4 ± 3.58	8/1/1′	0.014*
Mean red blood cell volume (fL)	83.6–98.2	90.1 ± 3.48	91.6 ± 3.20	7/3/0′	0.048*
Serum electrolytes					
Sodium (mEq/L)	138–145	140 ± 2.32	140 ± 1.89	5/2/3′	0.27
Potassium (mEq/L)	3.6–4.8	4.0 ± 0.25	3.95 ± 0.23	4/1/5′	0.56
Chloride (mEq/L)	101–108	104 ± 3.23	106 ± 2.80	9/0/1	0.004*
Blood urea nitrogen (mg/dL)	8.0–20.0	14.2 ± 5.76	13.8 ± 2.84	4/1/5′	0.81
Serum creatinine (mg/dL)	0.46–0.79	0.73 ± 0.19	0.75 ± 0.20	7/0/3	0.46
Uric acid (mg/dL)	3.7–7	5.46 ± 1.25	4.50 ± 0.93	2/0/8	0.014*
Urinary examination (n = 6)					
Urinary electrolytes					
Sodium (mEq/L)		159 ± 42.6	98.0 ± 35.1	0/0/6	0.02*
Potassium (mEq/L)		57.8 ± 24.4	57.8 ± 24.4	1/0/5	0.32
Chloride (mEq/L)		162 ± ± 59.3	104 ± 35.9	0/0/6	0.08
Neurohormonal test (n = 6)					
Adrenaline (pg/mL)	< 0.1	0.026 ± 0.024	0.029 ± 0.022	4/0/2	0.34
Noradrenaline (pg/mL)	0.1–0.5	0.27 ± 0.078	0.27 ± 0.09	3/0/3	0.95
Renin activity (ng/mL/h)	0.2–2.3	3.13 ± 3.13	5.67 ± 4.79	5/0/1	0.19
Aldosterone (pg/mL)	36–240	77.7 ± 12.7	94.7 ± 30.7	5/0/1	0.15
Anti-diuretic hormone (pg/mL)	< 2.8	1.33 ± 0.46	1.55 ± 0.89	3/0/3	0.66
Venous blood gas and metabolites (n = 6)					
pO_2_ (mmHg)	–	39.2 ± 13.6	52.3 ± 14.9	6/0/0	0.03*
Total CO_2_ (mEq/L)	–	28.3 ± 2.66	25.7 ± 1.71	0/0/6	0.01*
Actual HCO_3_^−^ (mEq/L)	–	26.9 ± 2.48	24.4 ± 1.62	0/0/6	0.008*
Acetoacetate (μmol/L)	13–69	41.8 ± 25.0	99.7 ± 45.4	6/0/0	0.02*
3-Hydroxybutyrate (μmol/L)	0–76	70.2 ± 38.8	200 ± 135	5/0/1	0.06
Total ketone bodies (μmol/L)	26–122	112 ± 63.5	300 ± 177	5/0/1	0.04*

*Statistically significant difference between before and after treatments (p < 0.05, paired *t* test)

## Discussion

Diabetic patients frequently present with electrolyte abnormalities [12]. The diuretic effects of SGLT2i may influence the serum chloride concentration, but few experimental [13–15] and clinical studies [16, 17] have deeply evaluated the effects and mechanistic consideration of SGLT2i on the serum chloride levels. The findings of the present study indicate that SGLT2i preserves or enhances the serum chloride concentration in T2DM/non-HF patients. Finding of regaining the serum chloride concentration under dosage of SGLT2i in the present study accords with the recent experimental studies [13–15]. Other findings of physical, hematologic, diabetic/metabolic, and neurohormonal tests were consistent with previous observations [1–3].

### Role of chloride and contribution of SGLT2i in regulating body fluid distribution

After sodium, chloride is the most abundant serum electrolyte [18], with a key role in the regulation of body fluids, electrolyte balance, preservation of electrolyte neutrality, and acid–base status, and is the essential component for assessing many pathologic conditions [19]. Recent studies [7–11] indicate that chloride, not sodium, is the primary electrolyte for regulating the fluid distribution in the human body according to the possible biochemical nature of the solutes, as follows. Solutes in the human body are classified as effective or ineffective osmoles on the basis of their ability to generate osmotic water movement, and osmotic water flux requires a solute concentration gradient [18]. “Tonicity” is the effective osmolality across a barrier, and regulates body water distribution to each body space compartment. According to the “chloride theory” [10], chloride ions, not sodium ions, are the key electrolyte for regulating the plasma volume in the human body. Thus, compared with cationic sodium ions, anionic chloride ions in the human body have the potential for “tonicity” in the vascular space [8, 10]; namely, chloride ions are the key electrolytes for primary regulation of the distribution of body fluid across body spaces, i.e., the intracellular, intravascular, interstitial compartments [18], and the pleural space [20].

As such, the present study demonstrated that SGLT2i could be classified as a “chloride-regaining” diuretic [10], such as acetazolamide [11, 21, 22] or vasopressin antagonists [23, 24]. “Chloride-regaining” diuretics, defined by the “chloride theory” [10], could have peculiar properties of preserving plasma volume and renal function [22, 25, 26], and the capability of draining interstitial body fluid by the serum chloride-associated enhancement of vascular “tonicity” [8, 10], as mentioned above. This concept is consistent with the recent clinical observations that SGLT2i reduces interstitial congestion without deleterious effects of arterial underfilling or predominantly decreased extracellular volume [27, 28].

### Underlying mechanisms of SGLT2i for regaining the serum chloride concentration

It is reasonable to consider that the increase in serum chloride concentration by the administration of SGLT2i is caused by its osmotic diuretic action with subsequent water excretion (aquaresis) and mild hemoconcentration, mechanisms similar to those of vasopressin antagonists [23, 24]. In addition, the findings of the present study suggest the following underlying mechanisms for regaining or enhancing the serum chloride concentration. First, reabsorption of urinary chloride is enhanced by activation of the renin-angiotensin-aldosterone system (RAAS) induced by SGLT2i due to blood pressure-lowering and blood vessel-contracting effects. Activation of the RAAS might be also induced by the reduced chloride supply to the macula densa by SGLT2 inhibition, similar to the actions of loop diuretics [29]. Controversies exist, however, about the effects of the SGLT2i on RAAS activity [30]. Indeed, a previous report pointed out that SGLT2i therapy inhibits RAAS activity [31]. It is likely that various levels of RAAS activity are observed in different phases (e.g., acute, sub-acute, or chronic) of SGLT2i treatment among heterogeneous T2DM patients [32]. The “chloride theory” [10] predicts that SGLT2i likely inhibits RAAS activity by its chloride-regaining effects [33]. In the present study, the RAAS activity at baseline was already slightly activated similar to a previous report [34], but RAAS activation was not markedly activated after SGLT2i treatment.

Second, it could be expected that a reciprocal increase in the serum chloride concentration might be caused by reducing the serum HCO_3_^−^ concentration [35] by buffering [36, 37] strong organic acid metabolites (e.g., ketone bodies of acetoacetic acid and 3-hydroxybutyric acid in the present study) under SGLT2i treatment [38, 39]. Lastly, inhibitory effects of SGLT2i on Na^+^–H^+^ exchanger 3 in the renal proximal tubules might reduce NaHCO_3_ reabsorption and concurrently enhance chloride reabsorption in the urinary tubules [40, 41]. According to these possible mechanisms, SGLT2i has a potential to preserve or enhance the serum chloride concentration. The precise mechanisms of SGLT2i for preserving the serum chloride concentration, however, require further exploration.

### Limitations

This study included a small number of patients at a single center, and there are important limitations to consider, including (1) a selection bias due to data availability and (2) low statistical power with wide variabilities in the data. Thus, the data must be cautiously interpreted. To my knowledge, however, it is the first study examined the effects and contributing factors of SGLT2i on the serum chloride concentration in T2DM/non-HF patients. Additional studies with more T2DM patients with or without HF, and an extended observational period are needed to better clarify the diuretic effects of SGLT2i on the chloride kinetics.

## Conclusions

An SGLT2i administration in T2DM/non-HF patients preserve or enhance the serum chloride concentration. Such a diuretic effect would preserve vascular volume and promote drainage of excessive extravascular fluid into the vascular space [27, 28] by enhancing vascular “tonicity” [8]. Caution is advised when using an SGLT2i in patients with hypernatremia/chloremia because these diuretics might lead to an increase in serum chloride and sodium levels [42].

## Data Availability

Not applicable. Conclusions of the manuscript are based on relevant data sets available in the manuscript.

## Abbreviations

HF: heart failure
RAAS: renin–angiotensin–aldosterone system
SGLT2i: sodium–glucose cotransporter 2 inhibitor
T2DM: type 2 diabetes mellitus
